# Attenuation of cGVHD by C5a/C5aR blockade is associated with increased frequency of Treg

**DOI:** 10.1038/s41598-017-03700-1

**Published:** 2017-06-15

**Authors:** Yulian Wang, Peilong Lai, Xiaomei Chen, Chang He, Xin Huang, Suxia Geng, Chenwei Luo, Suijing Wu, Wei Ling, Liye Zhong, Zesheng Lu, Peng Li, Jianyu Weng, Xin Du

**Affiliations:** 1 Department of Hematology, Guangdong General Hospital, Guangdong Academy of Medical Sciences, Guangzhou, Guangdong 510080 P. R. China; 2 State Key Laboratory of Ophthalmology, Zhongshan Ophthalmic Center, Sun Yat-Sen University, Guangzhou, 510060 P. R. China; 3 State Key Laboratory of Respiratory Disease, Guangzhou Institutes of Biomedicine and Health, Chinese Academy of Sciences, Guangzhou, Guangdong 510530 P. R. China

## Abstract

C5aR signaling plays an important role in the regulation of T cell activation and alloimmune responses in chronic graft-versus-host disease (cGVHD). However, direct evidence of this modulation and the efficacy of C5aR blockade in the treatment of cGVHD have not been demonstrated. We observed higher expression of C5aR on both monocytes and T cells of patients with cGVHD compared with healthy controls and non-GVHD patients after allogeneic hematopoietic stem cell transplantation. Our data also demonstrated a significant negative correlation between C5aR expression and regulatory T cells (Treg) frequency in cGVHD patients, indicating a potential role of C5aR in the generation and regulation of Treg. In addition, an *in vitro* experiment revealed C5aR deficiency promoted the development of Treg whereas C5a activation abolished the differentiation of Treg. Importantly, we found C5aR blockade by PMX53 attenuated the pathology of cGVHD and improved the survival of cGVHD mice. PMX53 had a direct regulatory effect on Treg commitment and increased TGF-β1 expression. Thus, C5aR signaling may induce and intensify cGVHD by down-regulating Treg induction. The modulation of C5aR activation by PMX53 may provide a potential therapy for cGVHD.

## Introduction

Chronic graft-versus-host disease (cGVHD) is the most common complication after allogeneic hematopoietic stem cell transplantation (HSCT), which has been widely used in the treatment of a large number of malignant and non-malignant hematological diseases^1, 2^. cGVHD is characterized by immune dysregulation in multiple systems and causes significant morbidity and mortality in 30–80% of individuals who survive for >100 days after HSCT^3^. Understanding of the basic biology of cGVHD has improved rapidly over the past decade. The donor T cells that attack the targeted host tissues and impair organ function are activated through innate and adaptive immune mechanisms in a complement pathway-dependent manner^2, 4, 5^. Complement is a key orchestrator at the interaction of antigen-presenting cells (APCs) and T cells^6, 7^ and the modulation of complement activation may provide a potential mechanism to regulate the response of donor T cells and the treatment of cGVHD. However, direct evidence of this modulation has not been demonstrated.

Complement is usually activated by one or more of distinct pathways - the classic, alternative, and lectin-binding pathways - in a cascade-like fashion^6^. The anaphylatoxins C3a and C5a are key effector molecules of the complement system that could play an important role in sensing and removal of pathogens and danger^8^. Recently, it has been shown that C3a and C5a can modulate adaptive immunity via interactions with their respective receptors on both innate and adaptive immune cells^9, 10^. C5aR is broadly expressed on a variety of cells, but particularly on T cells and myeloid cells like APCs. Its signaling is required for the effective antigen presentation from APCs to T cells and the subsequent modulation of T cell activation, differentiation and function^5, 10^. During cGVHD, APCs are activated in response to total body irradiation and present MHC-mismatched peptides, consequently initiate donor T cell activation, which has the capacity to attack the recipient^4, 11^. Given the crucial role of C5aR signaling in the induction and regulation of adaptive T cell responses C5aR may have the potential to induce and intensify cGVHD.

The reciprocal regulation of TGF-β, IL-6, IL-21 and IL-23 is affected by complement activation and could determine the differentiation and commitment of CD4+ T cells^12, 13^. C5a-C5aR-mediated signaling has been shown to contribute to the differentiation of Th1 and Th17 cells via the production of interleukin-12 (IL-12) by APCs and expression of the IL-12 receptor (IL-12R) by CD4+ T cells^14^. The absence of C5aR-mediated signals during T cell activation diverts naive T cells to a Treg response through TGF-β production^10, 15^. A new concept is emerging in which TGF-β and IL-6 induced the Treg differentiation through regulating C5a production and C5aR signaling activation, forming a positive feedback loop^16, 17^. Given the absence of Treg control of Th1 and Th17 cells in the pathology of cGVHD, the exact role of C5aR signaling in T cell phenotype in cGVHD needs to be clarified.

In this study, we found higher expression of C5aR on both APCs and T cells in patients with cGVHD and a negative correlation between C5aR expression and Treg frequency in cGVHD. Furthermore, the C5aR deficiency promoted the development of Treg, whereas C5a activation abolished the differentiation of Treg *in vitro*. Importantly, we found that C5aR blockade by mAb PMX53 attenuated the pathology of cGVHD and improved the survival of the cGVHD mice, accompanied with increased TGF-β1 expression and Treg generation. Thus, C5aR signaling may contribute to cGVHD through down-regulating Treg induction. We envisioned a novel therapy involving C5aR blockade by PMX53 for the treatment of cGVHD.

## Results

### Expression of C5aR is associated with the presence of cGVHD

**Figure 1:**
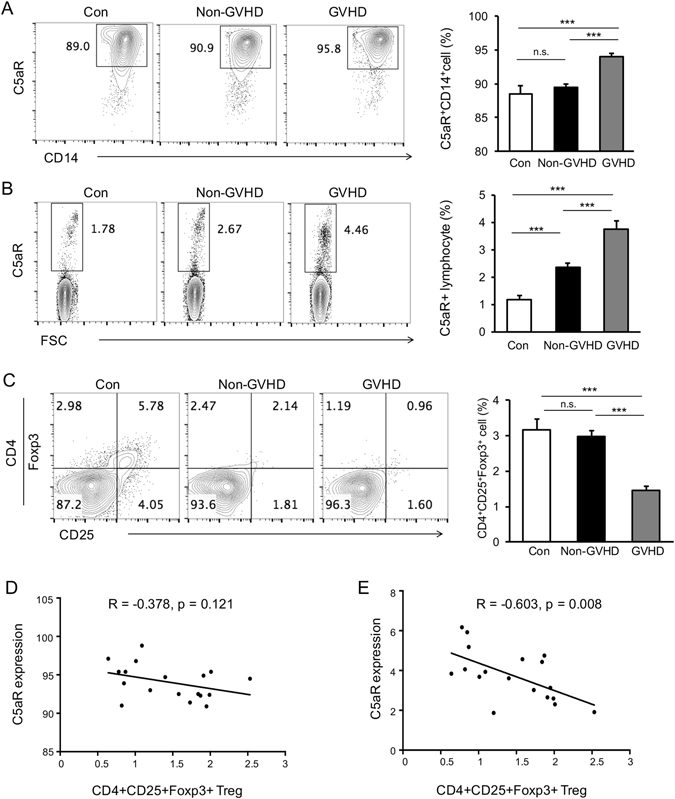
Expression of C5aR is increased and is associated with Treg reduction in cGVHD patients. (**A**) Representative flow cytometry plots showed C5aR expression in CD14+ monocytes from healthy controls, non-GVHD patients, or cGVHD patients. The statistical results revealed that C5aR expression was significantly up-regulated on the monocytes in cGVHD patients compared with healthy controls as well as non-GVHD patients who had undergone HSCT. (**B**) The lymphocytes were gated, and C5aR expression on these cells was also markedly increased in cGVHD groups. (**C**) Representative flow cytometry plots showed CD25 and Foxp3 expression in CD4+ cells from healthy controls, non-GVHD patients, or cGVHD patients. (**D**) Spearman’s correlation coefficient analysis revealed no significant correlation between C5aR expression on monocytes and the frequency of Treg, although the two parameters appeared to be negatively related. (**E**) There was a significant negative correlation between C5aR expression on lymphocytes and the frequency of Treg. *p < 0.05, **p < 0.005, ***p < 0.001.

To evaluate the potential relevance of the C5aR ligation during the course of cGVHD, we investigated the expression of C5aR on monocytes and lymphocytes in the blood of patients with cGVHD. The clinical characteristics of the patients and healthy controls are listed in Table 1. Peripheral blood mononuclear cells (PBMCs) were isolated from these patients, and the expression of C5aR examined on monocytes and lymphocytes was identified by flow cytometry on the basis of the distribution of FSC and SSC as well as the markers of CD14 and CD4, respectively. As shown in Fig. 1A, the expression of C5aR remained steady in healthy controls and non-GVHD patients who had undergone HSCT; however, C5aR was up-regulated on the monocytes in cGVHD patients and had significantly higher levels than those from healthy controls as well as non-GVHD patients. In addition, in order to evaluate the relevance of complement on T cell biology, the lymphocytes were gated, and the C5aR expression was also examined by FACS. As shown in Fig. 1B, the C5aR expression on lymphocytes increased in the cGVHD group compared to healthy control. These data suggested that the expression of C5aR on monocytes as well as lymphocytes is associated with the pathology of cGVHD.

**Table 1:** Clinical characteristics of the study population.

	Health Controls	Non-GVHD	cGVHD
Male to female ratio	6:5	9:6	12:6
Age	30.18 ± 5.81	31.06 ± 8.89	29.44 ± 7.97
Primary disease before HSCT	NA	ALL:5	ALL:8
		AML:8	AML:8
		CML:1	CML:2
		CMML:1	
Donor			
Sibling	NA	8	8
Matched unrelated	NA	7	10

Abbreviations: ALL = acute lymphoblastic leukemia; AML = acute myeloid leukemia; CML = chronic myelogenous leukemia; CMML = Chronic myelomonocytic leukaemia; NA = not applicable.

### Surface expression of C5aR inversely correlates with numbers of Treg

Next, we detected the frequency of Treg and evaluated the correlation between C5aR expression and Treg in the cGVHD patients. The CD4^+^CD25^+^Foxp3^+^ Treg cells are associated with a reduced risk of cGVHD and function to suppress autoreactive and alloreactive immune cells to reverse the established cGVHD^18^. As expected, the frequency of Treg was markedly reduced in cGVHD patients (Fig. 1C). Analyses of the Spearman correlation coefficient revealed no significant correlation between C5aR expression on monocytes and the frequency of CD4^+^CD25^+^Foxp3^+^ Treg, although two parameters appeared to be negatively related (Fig. 1D). A significant negative correlation was observed between C5aR expression on CD4+ T cells and numbers of CD4^+^CD25^+^Foxp3^+^ Treg (Fig. 1E). Thus, these data indicated that C5aR expression may be involved in Treg induction in cGVHD in clinical patients.

### C5aR deficiency promoted the development of Treg, whereas C5a activation abolished the occurrence of Treg *in vitro*

**Figure 2:**
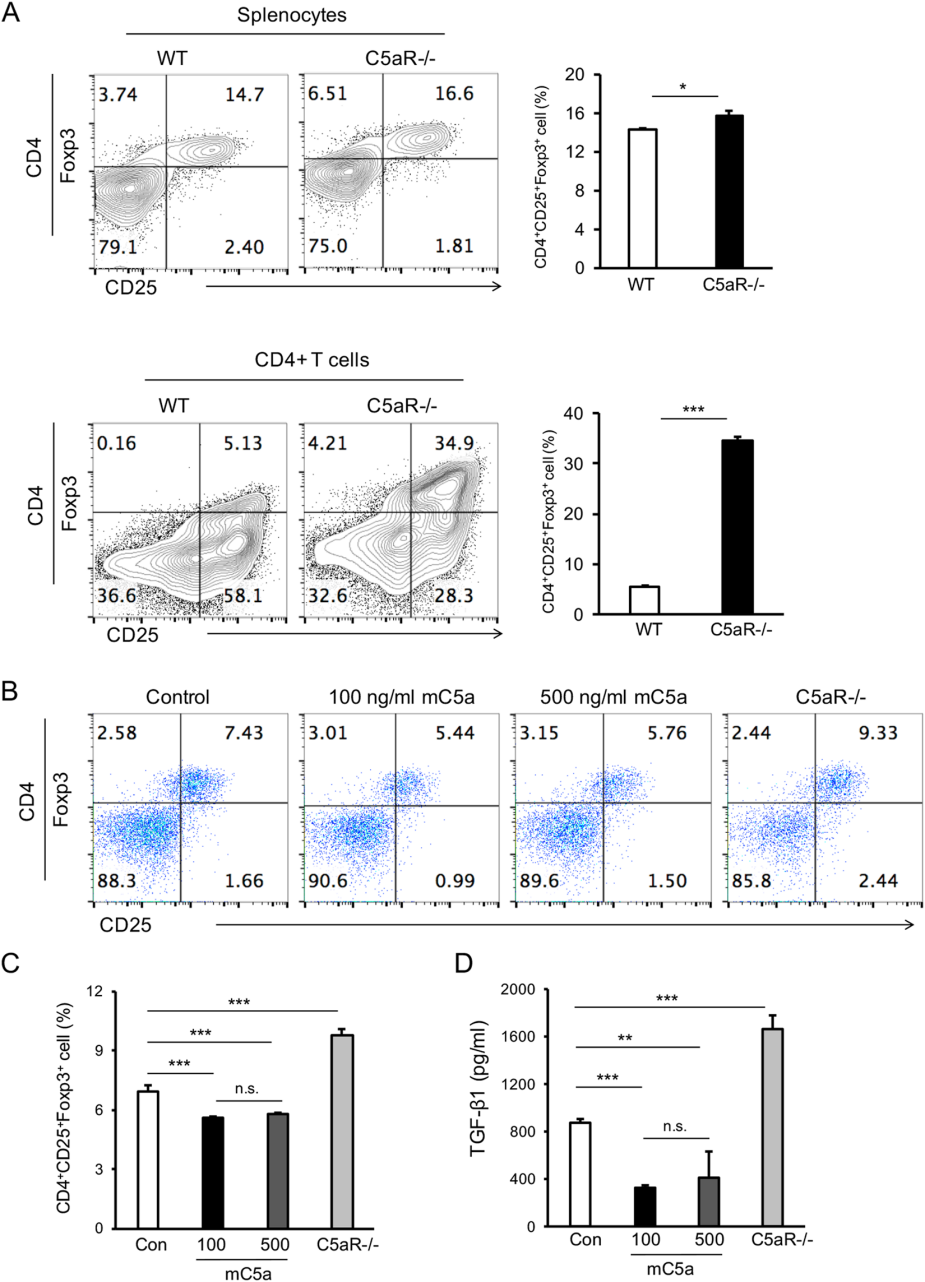
C5aR deficiency promoted the development of Treg, whereas C5a activation abolished the occurrence of Treg cell *in vitro*. (**A**, upper panel) Splenocytes from the C5aR deficient mice and WT mice were isolated and analyzed directly for the frequency of Treg. Representative flow cytometry plots showed CD25 and Foxp3 expression in CD4+ cells. (**A**, lower panel) CD4+ T cells from the C5aR deficient mice and WT mice were sorted and stimulated in the Treg polarization condition. Representative flow cytometry plots showed CD25 and Foxp3 expression in sorted CD4+ cells from WT and C5aR−/− mice. (**B**,**C**) The cultured splenocytes were stimulated with anti-CD3 and anti-CD28 antibodies for 3 days. The frequency of Treg was reduced in response to 100 ng/ml or 500 ng/ml mC5a stimulation. However, the splenocytes from the C5aR-deficient mice were activated and inclined to differentiate toward CD4^+^CD25^+^Foxp3^+^ Treg. (**D**) The supernatant protein level of TGF-β1 was decreased in the mC5a stimulation group, whereas it was significantly increased in the C5aR−/− splenocytes. At least five mice were used in flow cytometry experiment (N = 5) and there were at least three cell samples (N = 3) in (**B–D**); n.s. = no significance, *p < 0.05, **p < 0.005, ***p < 0.001.

To further detect the effect of C5aR signaling on the development of Treg, we first investigated the frequency of Treg in the isolated splenocytes from WT and C5aR deficient mice. Surprisingly, C5aR deficiency increased the frequency of CD4^+^CD25^+^Foxp3^+^ Treg in naïve splenocytes (Fig. 2A, upper) as well as purified CD4+ T cells cultured in Treg polarizing conditions (Fig. 2A, lower). In addition, in the cultured splenocytes stimulated by anti-CD3 and anti-CD28 antibodies, the development of Treg was significantly inhibited in response to C5aR activation by 100 ng/ml as well as 500 ng/ml RmC5a ligands. In contrast, the splenocytes from the C5aR deficient mice were activated and inclined to differentiate towards CD4^+^CD25^+^Foxp3^+^ Treg compared with WT-splenocytes (Fig. 2B,C). The supernatant protein level of TGF-β1, which has the ability to promote Foxp3 expression and maintain Treg function, was decreased after C5aR activation by RmC5a, whereas it was increased significantly when C5aR−/− cells were studied (Fig. 2D). These data suggested that C5aR activation inhibited the development of Treg *in vitro*.

### C5aR blockade by PMX53 attenuated the pathology of cGVHD *in vivo*

**Figure 3:**
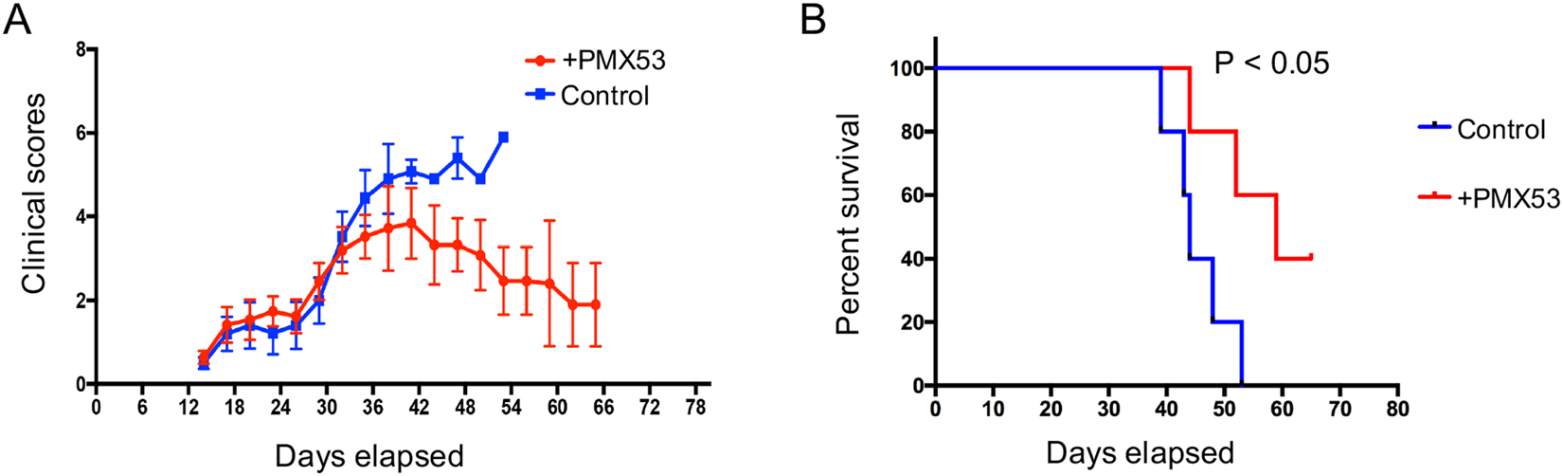
PMX53 treatment promoted the survival of cGVHD mice. (**A**) The clinical scores of cGVHD were reduced in the PMX53 treatment group, especially in the late stage of disease. (**B**) The survival rate data revealed that PMX53 treatment protected the cGVHD mice from death, and more mice lived longer. At least five mice were used in each group (N = 5).

**Figure 4:**
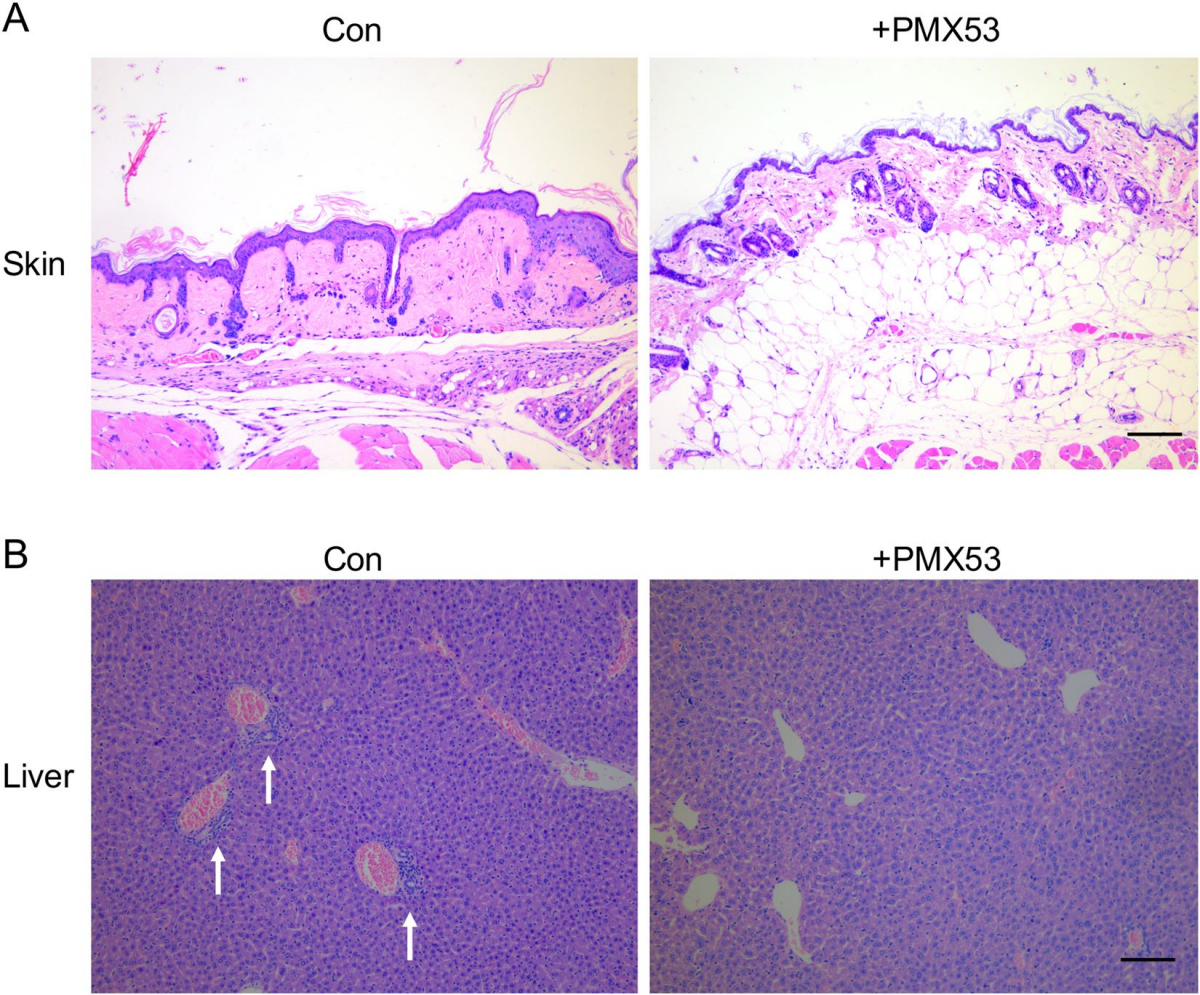
PMX53 treatment attenuated the pathological damage of cGVHD mice. (**A**) Skin histologic examination by HE staining showed that the cGVHD mice subjected to PBS treatment presented with damaged skin, including thickening in the epithelial layer, a loss of hair follicles, a lack of subdermal fat and heavy collagen deposition. However, the PMX53 treatment group exhibited a relative well-organized structure of skin compared to control cGVHD mice, including a reduced thickness of epithelial layer, more hair follicles, increased thickness of subdermal fat layer and decreased collagen deposition. (**B**) Liver histologic examination revealed clear perivascular infiltration surrounding the bile duct (white arrows) in the cGVHD mice, whereas few infiltrated cells were observed in the PMX53 treatment group. At least five mice were used in each group (N = 5). Scale bar: 200 μm.

Considering the potential role of C5aR signaling in Treg reduction and the incidence of cGVHD, we evaluated the effect of a cyclic peptide termed PMX53, a potent, highly selective C5aR antagonist, in treating cGVHD in animal model. Notably, the PMX53 treatment prevented the mice from exhibiting cGVHD, as evidenced by lower clinical scores of cGVHD (Fig. 3A) and higher survival rates (Fig. 3B). In addition, histological analysis by hematoxylin and eosin (H&E) staining displayed the pathological changes in multiple organs in the control cGVHD mice. Skin histologic examination revealed a thickening of the epithelial layer, loss of hair follicles and subdermal fat, ulcers in the epithelial and dermal layers, and heavy collagen deposition in the skin lesions of control cGVHD mice (Fig. 4A). In contrast, the PMX53 treatment group exhibited a relatively normal organizational structure of the skin, including a moderate thickness of epithelial layer and well-organized hair follicles. In the liver, perivascular infiltration was observed surrounding the bile ducts and extending into the parenchyma in the cGVHD mice. In contrast, PMX53 treatment resulted in lower pathology scores in the liver and little cellular infiltration (Fig. 4B). Together, our results suggest that C5aR blockade by PMX53 can ameliorate the severity of cGVHD.

### PMX53 treatment promoted the development of Treg and increased the accompanied expression of TGF-β1

**Figure 5:**
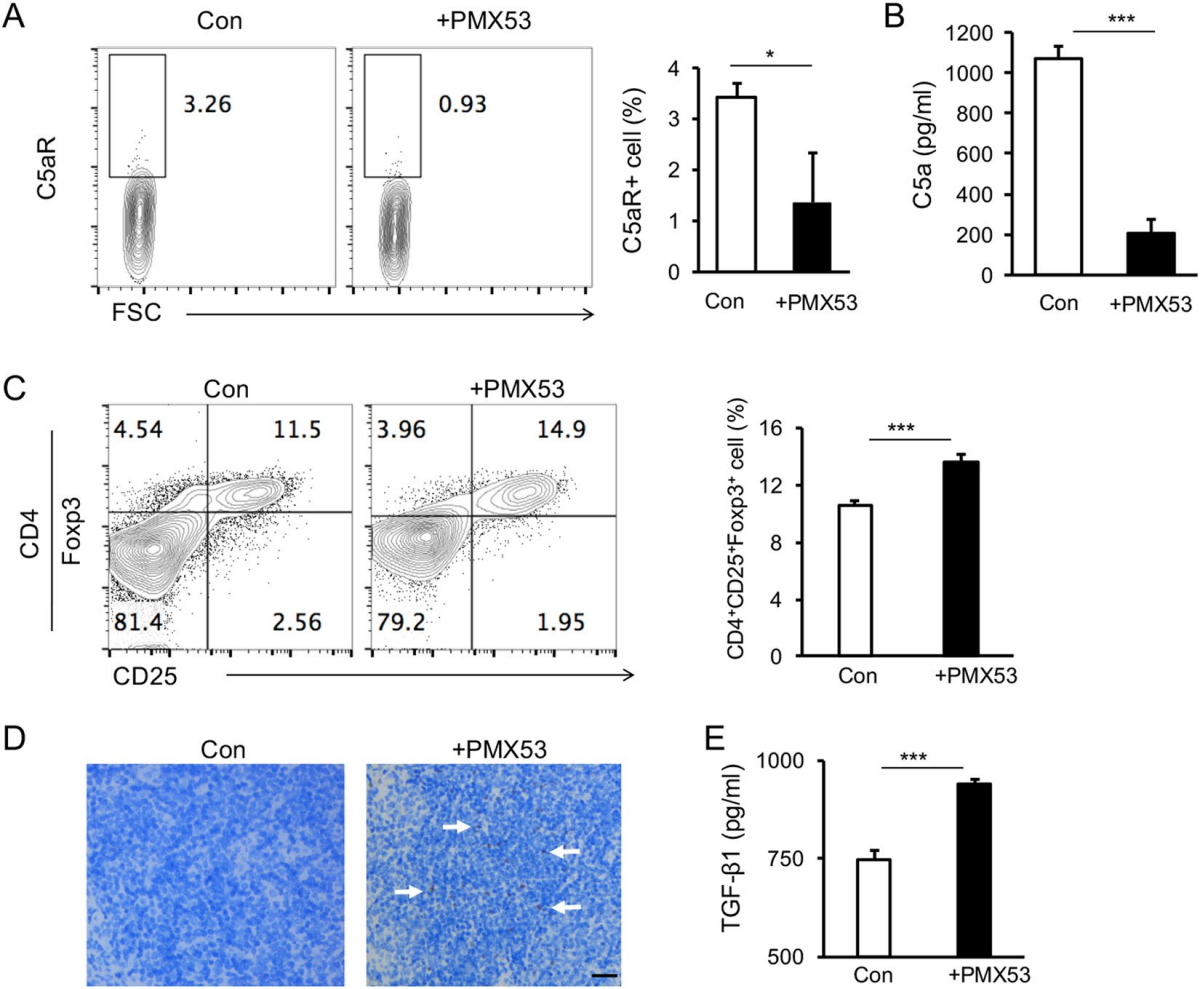
PMX53 treatment promoted the development of Treg and increased the accompanied expression of TGF-β1. (**A**) Representative flow cytometry plots showed C5aR expression in monocytes from cGVHD mice with or without PMX53 treatment. (**B**) ELISA result revealed that PMX53 treatment reduced the level of C5a in plasma from cGVHD mice. (**C**) Representative flow cytometry plots showed CD25 and Foxp3 expression in CD4+ cells from cGVHD mice with or without PMX53 treatment. (**D**) Expression of Foxp3, a key transcription factor in Treg, was increased in histological sections of spleen from cGVHD mice after PMX53 treatment. (**E**) PMX53 treatment also promoted the expression of TGF-β1 in the plasma in cGVHD mice compared with the control group. At least five mice were used in each group for flow cytometry and ELISA (N = 5) and three mice were used for histological analysis (N = 3), *p < 0.05, ***p < 0.001. Scale bar: 200 μm.

Mechanistically, we found PMX53 not only inhibited the presence of C5aR as well as C5a in cGVHD mice (Fig. 5A,B), but also up-regulated the frequency of CD4^+^CD25^+^Foxp3^+^ Treg (Fig. 5C) and Foxp3 expression in splenocytes (Fig. 5D) as well as TGF-β1 expression (Fig. 5E). These data suggested that C5aR blockade by PMX53 treatment may promote the development of regulatory cells, which would suppress the occurrence and severity of cGVHD.

## Discussion

The complement, a key orchestrator at the interface of innate and adaptive immunity, plays an important role in impairing immunomodulatory effects in cGVHD^19^. Here we demonstrated that C5aR blockade by PMX53 paves the way for T regulatory cell lineage commitment in cGVHD mice, highlighting an important role of the C5a-C5aR axis in the development and function of Treg, regardless of C5aR expressed on T lymphocytes or APCs. The cGVHD mice treated by PMX53 exhibited reduced clinical scores and prolonged survival time. In cGVHD patients, C5aR expression on monocytes was markedly increased, but did not show correlation with Treg numbers. However, the C5aR expressed on lymphocytes exhibited a negative correlation with Treg frequency in cGVHD patients. *In vitro* C5a stimulation suppressed Treg generation whereas C5aR deficiency promoted the TGF-β1-induced Treg commitment. These data suggested that pharmacologic targeting of C5aR would suppress cGVHD disease by enhancing the numbers of Treg.

A growing body of evidence demonstrates the central role of the complement system in shaping T cell responses^9, 20, 21^. Complement components and activators could be produced not only by APCs, including monocyte, macrophages, and dendritic cells, but also by T cells^22, 23^, indicating that complement is actively involved in the regulation of T cell effector immune responses^21^. There are two possible mechanisms^24, 25^. On the one hand, the complement system exert direct effects on T cells themselves; on the other hand, it affects T cell biology by regulating the function of APCs, which induce T cell priming, differentiation and trafficking^26^. In this study, we found that C5aR expressed on lymphocytes exhibited a negative correlation with Treg frequency in cGVHD patients, whereas the C5aR expressed on monocytes exhibited only a trend of negative correlation, suggesting that the complement participated in the regulation of Treg differentiation in cGVHD may be by C5aR expressed on T cells rather than APCs during their cognate interaction.

The complement system is crucial in modulating T lymphocyte responses^9, 26^. C5a-C5aR signaling has been established as a contributor to Th1 induction^27, 28^, consistently with an ‘expected’ pro-inflammatory function of complement. In addition, some data have suggested that C5aR signaling in dendritic cells is essential for biasing T cell differentiation into a Th17 response and also induces a pro-inflammatory response^29^. Interestingly, the C5aR activation plays an integral role in suppressing dominant immunologic tolerance^30^, which inhibits both Th1 and Th17 responses. Some studies reported that the C5a-C5aR signaling affects the modulation of Treg through suppressing the APCs generation of TGF-β1^9, 31^. Similarly, our work showed the suppressive effects of C5aR on Treg generation and cytokine production. We found that C5aR blockade augmented the production of TGF-β1, which plays an essential role in Treg commitment. Fitting nicely into this concept, some reports have recently revealed that when C5aR signals are not transduced and activated, the inductive TGF-β1 signaling initiates Foxp3+ CD4+ T regulatory cells. In turn, TGF-β1 could inhibit the C5a production, thereby suppressed the C5aR signaling. Given the fact that evolution of complement preceded that of the cytokine systems, it is conceivable that C5a participates in the differentiation of CD4+ T cells as well as the effects of cytokines.

PMX53, a molecule antagonist that specifically blocks the interaction of C5a with C5aR^32^, has undergone clinical trials for the potential treatment of inflammatory disorders, including rheumatoid arthritis and psoriasis. It has also been tested in mouse models for the treatment efficacy of periodontitis, sepsis, intracerebral hemorrhage, and other disorders^33, 34^. PMX53 could significantly reduce C5a-mediated inflammation in these diseases. However, it has not been shown to have the same effect in cGVHD. Our study established the cGVHD mouse model and evaluated the efficacy of PMX53 administrated by intraperitoneal injection every three days in cGVHD mice. Markedly reduced clinical scores as well as higher survival rates were observed in cGVHD mice with PMX53 treatment, indicating PMX53 may be a promising therapeutic candidate for cGVHD. Further, the increased generation of Treg in cGVHD after PMX53 administration suggested a potent suppressive role of PMX53 in attacking T effector cells in cGVHD through enhanced Treg generation and function.

In this study, we isolated the splenocytes from C5aR deficient mice and WT mice and found C5aR deficiency up-regulated the CD4+ CD25+ Foxp3+ T regulatory cells in the absence of any stimulation, suggesting that C5aR is involved in the maintenance of Treg in homeostasis. In addition, an increased frequency of Treg was observed in cultured C5aR−/− CD4+ T cells after 3 days of stimulation, thus suggesting a potential role of C5aR blockade in the modulation of Treg in activated CD4+ T cells. The resulting Treg exerted robust suppression of ongoing cGVHD disease. The adoptive supplement of Treg is a promising therapy; however, the obstacles of limited source and viability limit its use. The inductive effect of a lack of C5aR may be exploited as a potent method to generate Treg and suppress cGVHD diseases.

In conclusion, our data demonstrated that C5aR expression in cGVHD patients is significantly increased and is negatively correlated with Treg. We identified a critical role of C5aR in initiating aberrant effector T cell responses through impairing the regulatory T cell generation in cGVHD. Modulation of C5aR signaling by PMX53 may have a direct regulatory effect on Treg commitment, thus providing a means to ‘reset’ aberrant T cell responses. PMX53 requires more clinical investigation to expand its indications, such as non-classical inflammatory diseases including cGVHD.

## Methods

### Patients

As shown in Table 1, eighteen patients (12 Male, 6 female) with an average age of 29.44 ± 7.97 year-old who were diagnosed as cGVHD in the Guangdong General Hospital were enrolled in this study. The diagnosis of cGVHD was determined according to the NIH consensus criteria for cGVHD^35^. Fifteen patients without GVHD after HSCT were regarded as non-GVHD controls, and 11 healthy donors were considered as negative controls. The data of interest were as follows: gender, age, and primary diseases before HSCT. The Ethics Committees of Guangdong General Hospital approved the protocol (authorized number: GDREC2013061H) and all the participants provided written informed consent before enrollment. The study was performed according to the Declaration of Helsinki and the relevant ethical guidelines for research on humans.

### cGVHD mouse model and treatment

Ten- to 12-week-old B10.D2^H-2d^ (Jackson Laboratories, Bar Harbor, USA) and BALB/cJ^H-2d^ (Beijing Vital River Laboratory Animal Technology Co., Ltd, China) mice were used as donors and recipients, respectively. All the animal experimental design and procedures were reviewed and approved by the animal experimental ethics committee of Guangdong General Hospital (authorized number: GDREC2013061A). The experiments were conducted in accordance with protocols approved by the Institutional Animal Care and Use Committee of Sun Yat-Sen University. Recipient BALB/cJ mice received 700–850 cGy from a cesium irradiator and were reconstituted by tail vein injection with 8 × 10^6^ bone marrow cells with 8 × 10^6^ spleen cells from B10.D2 mice. The mice were monitored every 3 days for clinical score, body weight loss, and activities beginning at Day 14 after bone marrow transplantation (BMT). We evaluated the disease score based on the appearance of skin: healthy appearance = 0; skin lesions with alopecia less than 1 cm^2^ in area = 1; skin lesions with alopecia 1 to 2 cm^2^ in area = 2; and skin lesions with alopecia more than 2 cm^2^ in area = 3. Additionally, animals were assigned 0.3 point each for skin disease (lesions or scaling) on ears, tail, and paws with minimum score as 0 and maximum score as 3.9. When the established cGVHD model showed clinical scores above 0.6 at Day 29 after BMT, PMX53 (Tocris Bioscience, Minneapolis, USA) solubilized in PBS was injected intraperitoneally every three days at a dose of 1 mg/kg. The sequence of PMX53 is Ac-Phe-cyclo (Orn-Pro-D-Cha-Trp-Arg). Control mice received equal amounts of PBS injection. When the mice either died or were euthanized for humane reasons, the disease severity score at the time of death was included in subsequent mean scores.

### Histology

For histological assessment of cGVHD, representative samples from skin and liver were isolated and fixed in 4% formaldehyde and embedded in paraffin. Tissue sections of 6 μm were stained using H&E to study tissue damage in cGVHD with or without PMX53 treatment.

### Expression of Foxp3 by immunohistochemistry

The spleens were harvested from the mice and fixed in 4% paraformaldehyde and embedded in paraffin. For immunohistochemistry, the paraffin sections were cut into 5 μm sections and deparaffinized with xylene and rehydrated through graded alcohols. The antigen retrieval was carried out by heating the slides to 125 °C for 20 minutes. Then, the sections were digested with proteinase K (Dako) before incubation with peroxidase for 10 minutes. Sections were incubated with rabbit anti-Foxp3 primary antibody (diluted 1:200 in blocking buffer, Abcam) overnight at 4 °C. The sections were washed in washing buffer (0.1% Tween20 in PBS) and incubated for 1 hour at room temperature with the secondary antibody before development with 3,3-diaminobenzidine (DAB; brown product). The sections stained without primary antibody were negative controls. Images were acquired using a Zeiss microscope and used for further analysis.

### Splenocytes cultures and stimulation

C5aR−/− (C.129S4(B6)-C5ar1tm1Cge/J) mice and BALB/cJ wild-type mice were purchased from The Jackson Laboratories (Bar Harbor, ME). To generate the C5aR−/− mice, the embryonic stem cells with the mutation were injected into C57BL/6 blastocysts and then the chimeras were crossed to C57BL/6 females. The resulting heterozygote progeny were mated to C57BL/6 mice for 2 generations. At this point the mice were backcrossed 10 generations to BALB/c and got the homozygous. The splenocytes from these mice were isolated and erythrocytes were lysed. The splenocytes were cultured in RPMI-1640 (Gibco) supplemented with 10% fetal bovine serum (FBS, Gibco), 0.005% 2-mercaptoethanol (Gibco), 0.01% penicillin G and gentamycin (Sigma) at 37 °C, 5% CO2. The cell concentration was standardized to 3 × 10^6^ cells/ml and stimulated with IL-2 (40 ng/ml) (Peprotech), immobilized anti-CD3 (5 μg/ml) and soluble anti-CD28 (2 μg/ml) (eBioscience, USA) for 3 days. RmC5a (Cat#2150-C5-025, R&D System) at 100 ng/mL or 500 ng/mL was supplemented to activate the C5aR signaling. The supernatants were collected for TGF-β1 production via enzyme-linked immunosorbent serologic assay (ELISA), and cells were determined with flow cytometry. For the Treg polarization culture, the splenocytes from C5aR−/− mice and WT mice were isolated and the CD4+ T cells were enriched by negative selection using CD4+ T-cell isolation Kit (Miltenyi Biotec). The purified CD4+ T cells with >95% purity were stimulated at a concentration of 10^6^ cells/mL media in 96-well cell culture plates with plate-bound anti-CD3 (5 μg/mL), anti-CD28 mAb (2 μg/mL), IL-2 (40 ng/mL) and TGF-β1 (5 ng/mL) (Peprotech).

### Flow cytometry

C5aR expression in human PBMCs and cultured splenocytes were determined with multicolor flow cytometric analysis. Briefly, single cell suspensions were first blocked with Fc Block (BD Pharmingen, San Diego, CA) for 15 minutes and then incubated with antibodies for 20 minutes at room temperature. After being washed with buffer (PBS plus 1% BSA), the cells were analyzed with a FACSCanto II flow cytometer (Becton Dickinson, USA). Foxp3 intracellular staining was performed using an eBioscience kit (Cat#00-5521-00) according to the manufacturer’s protocol. Anti-human CD3 PE, Anti-human CD4 FITC, anti-human CD14 APC, anti-human CD25 APC, anti-human Foxp3 PE, anti-human C5aR PE, anti-mouse CD3 PE, anti-mouse CD4 FITC, anti-mouse CD14 APC, anti-mouse C5aR-APC, anti-mouse CD25 APC, and anti-mouse Foxp3 PE (eBioscience, USA) were used for these studies. The data were analyzed in FlowJo software (TreeStar).

### ELISA

TGF-β1 levels in plasma or supernatants of cultured splenocytes were assessed using enzyme-linked immunosorbent assay (ELISA kit, eBioscience, USA) according to the manufacturer’s instructions. C5a expression in plasma was also detected by ELISA (ELISA kit, R&D Systems, USA). Samples were detected in triplicate relative to standards supplied by the manufacturer and analyzed for significant differences among different groups.

### Statistical analysis

Statistical analysis was performed with SPSS software version 13.0 (Inc., Chicago, IL, USA). Group comparisons of flow cytometry data and ELISA data were analyzed with Student’s t-test or One-way analysis of variance. The nonparametric tests were also used to analyze and confirm the data. Bivariate correlation analysis was performed to determine the relationship between the expression of C5aR and the frequency of CD4+ CD25+ Foxp3+ T cells in cGVHD. Survival curves were plotted as Kaplan-Meier curves and analyzed with log-rank tests. A P value < 0.05 was considered significant.
